# Unlocking the Potential of the Superficial Cervical Plexus Block in Chronic Pain Management: A Narrative Review and Single-Center, Retrospective Case Series

**DOI:** 10.3390/jcm13216310

**Published:** 2024-10-22

**Authors:** Joe Zako, Jordi Perez

**Affiliations:** 1 Faculty of Medicine, Université de Montréal, Montreal, QC H3T 1J4, Canada; joe.zako@umontreal.ca; 2 Department of Anesthesia, McGill University, Montreal, QC H3A 0G4, Canada

**Keywords:** superficial cervical plexus, nerve block, chronic pain, cancer pain, interventional pain

## Abstract

**Background/Objectives**: The anesthetic block of the sensory branches of the superficial cervical plexus renders a specific area of the face, head, and anterior neck insensible and painless. Chronic pain in these areas can be difficult to diagnose and treat. In this report, we briefly review the existing evidence on the topic of the superficial cervical plexus block (SCPB) to set the context for our research. We then share our own clinical experience with the SCPB for managing chronic pain syndromes from both cancerous and non-cancerous etiologies. **Methods**: We first performed a comprehensive literature search and narrative review of clinical cases and studies that utilized the SCPB as an analgesic technique. We then conducted a retrospective case series of all patients who had received an SCPB at our pain clinic since 2020. **Results**: Our literature review found only a few cases reported, with most of them focusing on acute painful emergencies and perioperative pain syndromes and only very few addressing chronic pain. In our pain clinic, 14 patients received one or more SCPBs for chronic pain management. In 43% of these cases, the pain was related to cancer. The most common areas of pain corresponded to the regions supplied by the transverse cervical and great auricular nerves. The procedures were uneventful in all cases, and patients rated them as effective and worthwhile 71% of the time. **Conclusions**: Despite the lack of high-quality studies on SCPBs in pain management, the authors’ experience suggests that it is a valid minimally invasive alternative for managing chronic face, head, and neck pain.

## 1. Introduction

### 1.1. Pain in the Face, Head, and Neck

The assessment and treatment of chronic pain arising from facial, head, and anterior cervical structures are particularly challenging; this can be explained by the multiple possible sources of pain and their complex neural interconnexions. The complexity of managing chronic pain in these patients is matched by its broad range of differential diagnoses that must be considered, including cranio-mandibular disorders, headaches, facial neuralgias, and head and neck cancers [1]. A multidisciplinary approach to assess and manage these chronic pain syndromes may facilitate an accurate diagnostic and successful selection of analgesic treatment.

### 1.2. Anesthetic Blocks for Chronic Pain Management

Chronic pain is usually defined as any pain lasting for a period longer than 3 months [2]. Despite clinical recommendations, the mainstream management of chronic pain remains centered around long-term pharmacological options, including opioids. In these cases, guidelines emphasize the necessity of a multidisciplinary approach to assessment and management in order to achieve better outcomes and prevent adverse consequences owing to the prolonged use of systemic medication [3].

Among the recommended opioid-sparing multidisciplinary approaches, interventional pain procedures may be considered in cases when the pain is restricted to a confined anatomical area potentially innervated by a few easily identifiable nerve structures. Regional blocks can be effective in the management of pain because they interrupt nociceptive flow from its source, hence helping not only with the diagnosis but also with the management of this pain [4]. The superficial cervical plexus block (SCPB) is a relatively simple anesthetic technique used in various head and neck surgeries that interrupts the sensory message from selected anatomical areas of the face, the head, and the anterior aspect of the neck [5]. Its indications and effectiveness for the management of chronic pain are poorly described.

### 1.3. Purpose of This Study

The purpose of our study was to present a cohesive analysis combining our clinical experience with the SCPB in chronic pain management and a narrative review of the existing literature on the topic. We aimed to integrate our own clinical findings with a comprehensive review of scientific data regarding the indications and outcomes of the SCPB for pain management, offering a more thorough understanding of the SCPB’s effectiveness and applications.

## 2. Materials and Methods

### 2.1. Literature Review

We conducted an exhaustive literature search in PubMed, Scopus, and Embase (Elsevier) for articles and reviews published in English with the following keywords: “pain”, “chronic pain”, “acute pain”, “head and neck pain”, “cancer pain”, “interventional pain”, “pain management”, and “superficial cervical plexus block”.

### 2.2. Case Series

#### 2.2.1. Patient Population and Data Collection

Our institution’s ethics board approved this research, which is a retrospective chart review conducted at a single center by a single physician. This study involves patients from the Alan Edwards Pain Management Unit and the Cancer Pain Clinic at McGill University Health Centre who received one or more SCPBs for managing their chronic pain.

The charts analyzed were selected from the interventional list of the attending physician (J.P.) who performed these procedures. The cases included patients who had received an SCPB for chronic pain management since 2020.

The data collected from patients’ electronic medical records included sex and age, pain diagnosis, and pain severity prior to the procedure. Pain severity was recorded in two formats: some charts classified it as Mild, Moderate, or Severe, while others used a numerical rating scale (NRS), with scores categorized as <4/10 for Mild, 4/10 to 7/10 for Moderate, and >7/10 for Severe. Overall perceived benefits were assessed as satisfactory if there was a decrease in at least one pain category (e.g., from Severe to Moderate or from Moderate to Mild). Additionally, the data included pharmacological analgesic methods, specifics of the blocks performed, any complications, post-block treatment options, and the need for repeat blocks.

#### 2.2.2. Superficial Cervical Plexus Block Technique

All procedures were performed in our institution’s interventional rooms and all patients were discharged home after their procedure. Before a block, patients were briefly interviewed to review their case, re-explain the procedure, address questions, and obtain written informed consent. The technique was performed under sterile conditions with ultrasound (US) guidance and the patients laying in a lateral decubitus or supine position, fully monitored (a more detailed description of the block technique is provided in the brief narrative review below).

## 3. Brief Narrative Literature Review

### 3.1. Anatomical Description

The cervical nerve plexus originates from the ventral rami of nerve roots C1 to C4 to produce deep and superficial branches. Deep muscular branches supply the infra-hyoid and sub-occipital muscles of the neck as well as the diaphragm through the phrenic nerve. The superficial (or cutaneous) branches split off externally to the prevertebral fascia [5] to emerge near the midpoint of the posterior border of the sternocleidomastoid muscle (SCM) and fan out into four individual nerves. The first and most rostral is the lesser occipital nerve (LON) supplying the skin of the upper neck and retro-auricular scalp. The great auricular nerve (GAN) supplies the ante-auricular lateral aspect of the face. The transverse cervical nerve (TCN) supplies the skin of the anterior triangle of the neck. The supraclavicular nerve (SCN) supplies the skin over the clavicle, as well as the lateral neck, the shoulder, and the upper pectoral region [6].

### 3.2. Indications for SCPB for Acute and Chronic Pain

The superficial cervical plexus block (SCPB) is a commonly performed anesthetic technique for surgical procedures such as carotid endarterectomies, thyroidectomies, parathyroidectomies, and clavicle fracture repairs [7,8,9]. However, the usage and effectiveness of SCPBs in the context of pain management have almost exclusively been reported in case reports and short case series.

For the sole purpose of pain relief, our literature review found that the SCPB seems to mostly be performed in the emergency department, and the most frequent indication for this procedure is the management of acute clavicular fracture pain [10,11,12]. Other conditions that have benefitted from the SCPB in an emergent context include paracervical muscle spasms, rotator cuff injuries, acromioclavicular joint separations, radicular or neuropathic neck pain, and acute herpes zoster pain [10,13,14,15].

For the management of chronic pain, the indications found in the literature include chronic post-whiplash headaches, post-herpetic neuralgia, and referred somatic cervical pain [16,17,18,19]. Other possible indications for an SCPB for chronic pain management are not well documented. However, it is worth noting that the block and neurolysis of selected terminal branches of the SCP, specifically the LON and the GAN, have been thoroughly described [20,21].

In the context of cancer-related procedures, the use of the SCPB has shown promise in various surgical interventions, including unilateral thyroid lobectomies and tracheal dissections for thyroid cancer, as well as mastectomies for breast cancer; its application has demonstrated effectiveness in reducing the need for post-operative analgesia and enhancing the quality of recovery [22,23,24]. However, the literature on the use of SCPB for chronic, cancer-related pain is notably limited. One case report by Kim H. et al. describes the usage of the SCPB in the relief of a brain cancer metastasis-related cervicogenic headache; in this case, the mass was situated in the left anterior neck and, after the procedure, this patient’s pain subsided for a week before there was a need for an additional block [25]. Additionally, in a study by Nasir et al., a cohort of patients with head and neck cancer who presented with neuropathic pain arising from the three trigeminal nerve divisions or from the superficial cervical plexus received interventional treatments (such as trigeminal blocks or SCPBs); the authors reported successful pain management in 85% of these cases [26].

### 3.3. Technical Description

The patient lies either on a lateral decubitus or supine with the head turned contralateral to the side of the block. Due to the vital importance of neighboring anatomical structures, it is recommended by US guidance to identify the target, navigate the needle, and control the spread of the injectate [5,7]. It is worth noting that studies have shown that there is no significant difference in success rate between landmark-based and US-guided techniques in terms of short-term anesthetic results [27]. However, in the field of interventional pain medicine, authors recommend following US guidance to increase anatomical accuracy by using the least amount of local anesthetic [5,7].

**Figure 1:**
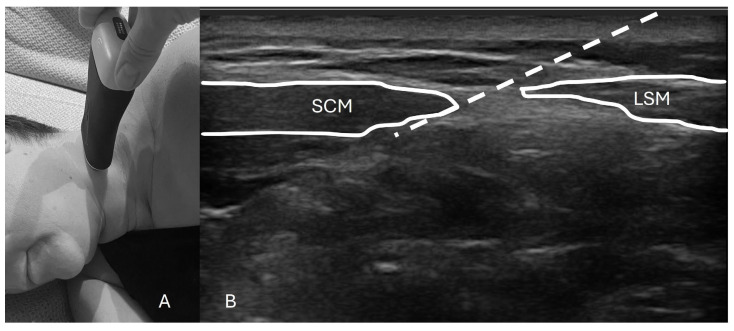
Probe placement and ultrasound image with landmarks. (**A**) The patient is positioned in the lateral decubitus position with the ultrasound probe placed approximately at the middle third of the sternocleidomastoid muscle (SCM). (**B**) An axial ultrasound scan of the cervical area shows the SCM and levator scapulae muscle (LSM). The white striped line indicates the needle trajectory, with the final dot marking the anatomical target for the injection.

To perform this technique, one must first identify the posterior border of the sternocleidomastoid (SCM) muscle extending from the mastoid process (most cranial insertion of the SCM) all the way down to the lateral projection of the C6 vertebrae. The US probe should provide an axial cut of the neck at the midpoint of the line of the SCM to a height close to the cricoid cartilage. With an “in-plane” approach, the needle is inserted and lands no more than 1 to 2 cm antero-medially under the SCM (Figure 1). After negative aspiration, a volume of local anesthetic is deployed underneath the SCM anterior to the levator scapulae muscle while being careful not to pierce through the prevertebral fascia [5,7]. Figure 1 shows an example of a typical setup for this procedure.

Our literature review found that the most commonly used local anesthetic was bupivacaine at various concentrations (0.25 and 0.5%) [5,10,11,12,16]; ropivacaine and levobupivacaine are also used [5,13,17,18]. Some authors even utilize lidocaine despite its short-lasting effects [14,15,16]. In some cases, adjuvants were added to prolong the effect, such as epinephrine, triamcinolone, and methylprednisolone [10,13,14,15,16,17,18]. Regardless of the type of local anesthetic used, the vast majority of volumes injected seem to vary between 5 and 10 mL, with only very few cases of injecting slightly less than 5 mL or between 10 and 15 mL [5,10,11,12,13,14,16,17,18].

### 3.4. Contraindications, Side Effects, and Potential Complications

The are very few specific contraindications for the SCPB. The most frequently mentioned concern either patients with paralysis of the contralateral phrenic nerve and diaphragm, patients with previous neck procedures including surgery or radiation, or patients with an infection at the planned injection site [5,7].

The potential complications associated with the SCPB procedure are also relatively infrequent. The most commonly reported can be attributed to the spread of the injectate into deep cervical plexuses and surrounding nerves. Paralysis of the ipsilateral phrenic nerve is the one most commonly reported [5]. There is also a possibility of recurrent laryngeal nerve paresis, deep cervical and even brachial plexus blockade, and accessory nerve dysfunction, all of which can cause weakness in the associated muscles [7]. There is one case report of temporary ipsilateral Horner’s syndrome after an SCPB that self-resolved after 90 min; the authors recommended limiting the anesthetic volumes while ensuring appropriate needle placement and avoiding excessively deep needle insertion to prevent these complications [12].

US-guided techniques represent a more secure option than the landmark-guided intervention if the goal is to avoid complications, especially if the physician is less familiar with the procedure. Additionally, the anatomical accuracy allows for reduction of injectate, thus reducing unwanted neighboring nerve blockades. Other potential complications include infection, hematoma, accidental jugular vein or carotid artery puncture, and local anesthetic systemic toxicity if the anesthetic enters the bloodstream [7].

### 3.5. Outcomes

The main outcome in much of the literature on the topic of the SCPB in pain management relates to pain relief after the procedure; the principal outcome measuring tools are subjective pain scales with various ranges.

In general, patients who undergo an SCPB seem to experience improvement of perceived pain, with only very rare neutral or negative outcomes. The timeframe for assessing post-block pain is not the same among all publications; some authors only assessed the results immediately after the SCPB procedure, while others followed up with their patients on a longer-term basis to assess pain recurrence or complications.

In some cases, there has been a necessity to repeat the procedure more than once to obtain more significant, durable, and prolonged pain relief.

It is also important to note that there also exist cases where clinicians have combined the SCPB with other types of blocks, such as a stellate ganglion block, which is known to alleviate what is called sympathetically mediated pain [13].

Complications of the SCPB might be rare, but they are not unseen. Fortunately, most cases in the literature either resolve spontaneously or are globally asymptomatic (in the case of hemidiaphragm paresis in an individual with no prior respiratory dysfunction, for example).

There are a few interesting facts to take away from the current literature on the topic:

1. A case series of patients receiving this block in the emergency department reported that it seemed to be slightly less effective in the alleviation of rotator cuff pain; they hypothesized that this was due to the hybrid innervation of the shoulder area by both the superficial cervical plexus and the brachial plexus, in addition to a certain degree of anatomical variation in nerve distribution and location [10].
2. A prospective double-blind clinical trial found that preoperative SCPB for patients undergoing tympano-mastoidectomy was successful in significantly reducing post-operative pain as well as opioid consumption when compared with the control group [28].
3. A case of a patient with facial herpes zoster receiving an SCPB reported success in the management of the acute pain yet failure to prevent onset of post-herpetic neuralgia in the same way deeper epidural or paravertebral blocks would [15]. However, an anatomical study by J J Pandit et al. shows that superficial cervical plexus injections tend to spread to the deep space through the porous prevertebral fascia [29]; therefore, there remains the theoretical possibility that the SCPB could be useful in preventing post-herpetic neuralgia, especially with larger quantities of injectate (which come with a slightly increased risk of developing the complications listed above).

## 4. Clinical Results

### 4.1. Overview

Fourteen charts were identified for further analysis. Demographic data, diagnosis, and details about the cases and the procedures can be found in Table 1 and Table 2. Of our patients, 43% received an SCPB for the treatment of chronic pain caused by head and neck cancer or its treatment.

### 4.2. Case Characteristics

#### 4.2.1. Pain Distribution and Intensity

The pain distribution of patients undergoing the procedure corresponded to one or more of the four superficial branches of the SCP: the great auricular nerve (GAN), lesser occipital nerve (LON), transverse cervical nerve (TCN), and supraclavicular nerve (SCN). A description of the anatomical areas supplied by each of these nerves is available in the literature review above. Among the patients treated, the most targeted areas were the ones supplied by the TCN (eight cases) and the GAN (seven cases). The least commonly targeted areas were the ones supplied by the LON (two cases) and the SCN (two cases).

Among patients with cancer-related pain, the most frequent painful area was the one supplied by the TCN (five out of six cases). In contrast, among non-cancer patients, the most frequent painful area was the one supplied by the GAN (four out of eight cases).

All patients reported at least moderate baseline pain. Patients with both moderate and severe baseline pain reported satisfactory pain relief at follow-up. However, the only two patients reporting unsatisfactory pain relief had severe baseline pain.

#### 4.2.2. Procedure Specifics

The main local anesthetic used was bupivacaine (10/14 cases) and the most frequent volume used was 5 mL (11/14 cases), which is on the lower end of the usual volume range used for this type of procedure.

#### 4.2.3. Clinical Outcomes

After the procedure, two patients from the non-cancer pain clinic were lost to follow-up; for analytical purposes, these cases were considered negative outcomes.

Additionally, two other patients—one from the cancer pain clinic and one from the non-cancer pain clinic—reported a lack of effect and the procedure was deemed to have failed. We could not find any pre-procedure characteristic suggesting a link to negative or positive outcomes after the block.

Overall, 10 of the 14 cases (71%) reported satisfactory pain relief at their follow-up visit, with these outcomes being equally distributed between the cancer clinic and the non-cancer clinic patients. There was no meaningful difference between the targeted nerve in successful cases versus those that failed. In total, 4 cases among the 10 that reported successful pain relief requested repeated procedures. No immediate or long-term complications were documented for any of our cases.

Of the total 12 patients that were not lost to follow-up, 6 people saw their pharmacological treatments after the block remain unchanged, while 3 saw the intensity of their analgesic treatment decrease, and 3 others saw it increase. Both patients with unsatisfactory pain relief were part of the subgroup of patients that did not change their pharmacological treatments after the block.

## 5. Discussion

This retrospective study examines a small clinical sample of patients undergoing a specific interventional technique for managing chronic pain of various etiologies. Although the preliminary results suggest apparent efficacy and safety, the research lacks sufficient rigor to provide robust scientific evidence to lead to strong clinical recommendations. This study has several weaknesses: the sample size is too small to draw reliable analytical conclusions; data were collected retrospectively from patient charts, raising concerns about data accuracy and interpretation; outcomes were not compared to a control group that did not undergo the procedure, making it difficult to assess whether the SCPB is more effective than alternative treatments; and the data only offer a snapshot of outcomes immediately or shortly after the procedure, with no information on the long-term duration and quality of the analgesic effect, hence the impossibility of drawing correlations between pre-procedure status and success of the block.

Although it is difficult to make any meaningful conclusions from our data, this research remains valuable for several reasons. First, the technique is both easy to perform and safe. Second, interventional options serve as valid adjuncts for managing particularly challenging, drug-resistant chronic pain syndromes. Lastly, addressing drug-resistant cancer pain requires exploring any available effective alternative to achieve effective and prolonged pain relief. We acknowledge that the benefits brought on by the SCPB in these cases should not be viewed as an absolute success in managing chronic pain. We believe that interventional pain management is just one of many analgesic tools that should be considered as part of a multidisciplinary approach to pain management and should very rarely be offered in isolation.

In patients presenting with cancer-related symptoms, the negative impact that pain can have on their quality of life and even their overall survival is well documented [30].

It is important to highlight the value of minimally invasive interventional techniques as adjuvants to multidisciplinary pain strategies.

Most interventional techniques reported for managing peripheral chronic pain originate from the field of locoregional anesthesia. These procedures have demonstrated benefits in managing intraoperative and postoperative pain, leading to a reduced need for systemic analgesics, improved postoperative comfort and decreased incidence of chronic postoperative pain [22,23,24,28,31].

The SCPB for chronic pain management can be performed as an outpatient procedure, requiring only a short period of post-procedural observation. This block can be carried out in any clinical setting, provided that basic safety precautions are followed to ensure the well-being and comfort of both patients and clinicians. The necessary equipment is relatively ubiquitous and inexpensive, including basic sterile materials, local anesthetics, and a portable ultrasound scanner.

Patients often experience immediate pain relief from the SCPB, which confirms the procedure’s effectiveness and helps to reassure them that the targeted nerve plays a role in their chronic pain. This quick relief can also boost their overall confidence in the treatment, potentially enhancing their perception of its benefits, especially in the case of repeated block administrations. As a result, some patients choose to reduce or stop using other systemic pain medications. The reduction in medication and the decrease in pain contribute to a better overall quality of life and greater satisfaction with the treatment. Currently, there is no widely accepted explanation for why an injection of a short-acting local anesthetic provides analgesic benefits beyond its expected duration. Several hypotheses attempt to explain this phenomenon. Some researchers suggest that peripheral pain inputs contribute to maintaining central sensitization and allodynia, and that blocking these inputs can cut this cycle, leading to pain relief [32]. Others propose that even temporary pain relief allows for mental and functional rehabilitation, resulting in lasting pain relief after the effects of the block wear off [33]. Finally, the placebo effect is another possibility, as many studies lack control groups to verify their findings. In some cases, as shown in our results, multiple blocks are needed to provide adequate, long-lasting relief.

In conclusion, while this article fails to strengthen the scientific evidence supporting the use of SCPB for chronic pain management, it does show that the procedure was safe and effective for our specific group of patients with moderate to severe pain. To further validate our hypothesis that the SCPB may be a viable and potentially superior alternative to systemic medications for managing severe, drug-resistant pain in the anterior neck and head—particularly in patients with chronic conditions such as cancer—more rigorous comparative studies are needed.

## Figures and Tables

**Table 1:** Patient demographics and pain characteristics.

Patient #, Sex, and Age	Pain Diagnosis			Pain Location (Nerve Area)	Initial Severity	Initial Treatment
	Cancer-Related?	If Cancer-Related				
		Treated?	Treatment-Related?			
1—Male, 58	Yes (non-Hodgkin’s lymphoma)	Yes	Yes	TCN + SCN	Moderate to severe	Topical NSAIDs
2—Female, 56	No (chronic cervicogenic headaches)			LON	Moderate to severe	Unknown
3—Female, 70	Yes (SCC mandible)	Yes	Yes	GAN + TCN	Unknown	APAP, GBP
4—Female, 67	Yes (SCC gingival)	Yes	Yes	GAN + TCN + SCN	Moderate to severe	Opioids, GBP
5—Female, 44	No (post-herpetic neuralgia)			GAN + TCN	Severe	TCA, GBP, SNRIs, NSAIDs, APAP
6—Male, 63	No (first-bite syndrome, parotid pain, treated oropharyngeal tumor)			GAN	Severe	Opioids, NSAIDs, TCA, GBP
7—Female, 30	No (great auricular neuralgia)			GAN	Moderate	Unknown
8—Male, 44	Yes (SCC lateral tongue)	Yes	Yes	GAN	Moderate	NSAIDs, opioids, APAP, GBP
9—Female, 75	No (post-cyst removal pain behind the ear)			LON	Severe	Opioids, BZD, NSAIDs
10—Female, 29	No (cervical spinal stenosis, spine surgery, SCM tension)			TCN	Severe	Interventional, physiotherapy, SNRIs, GBP, TCA, opioids
11—Male, 45	No (post-frostbite great auricular neuralgia)			GAN	Moderate	GBP, NSAIDs, interventional
12—Male, 80	Yes (SCC gingival)	Yes	Yes	TCN	Severe	GBP, TCA
13—Female, 64	Yes (SCC tonsil)	Yes	Yes	TCN	Severe	Opioids
14—Female, 44	No (post-thyroidectomy)			TCN	Severe	GBP

Abbreviations: # = Patient sequential identification number; APAP = Acetaminophen; BZD = Benzodiazepines; SCC = Squamous cell carcinoma; GBP = Gabapentinoids; GAN = Great auricular nerve; LON = Lesser occipital nerve; NSAID = Non-steroidal anti-inflammatory drugs; SCM = Sternocleidomastoid muscle; TCA = Tricyclic antidepressant drugs; TCN = Transverse cervical nerve; SCN = Supraclavicular nerve; SNRI = Serotonin and noradrenaline reuptake inhibitors.

**Table 2:** SCPB specifics and post-block follow-up.

Patient #, Sex, and Age	Block Specifics	Complications	Post-Block		Post-Block Pharmacological Treatment	Repeat Blocks	
			Follow-Up Time	Satisfactory Pain Relief		Needed?	How Many?
1—Male, 58	Right, bupivacaine, 3 mL	None	4 months	Yes	Unchanged	No	
2—Female, 56	Right, lidocaine, 5 mL	None	6 months	Yes	Unchanged	No	
3—Female, 70	Left, lidocaine, 5 mL	None	1 month	Yes	Increased	Yes	3
4—Female, 67	Right, lidocaine, 5 mL	None	1 month	Yes	Decreased	Yes	2
5—Female, 44	Left, bupivacaine, 5 mL	None	Loss of FU	Loss of FU	Loss of FU	N/A	
6—Male, 63	Left, bupivacaine, 5 mL	None	Loss of FU	Loss of FU	Loss of FU	N/A	
7—Female, 30	Left, bupivacaine, 4 mL	None	7 months	Yes	Unchanged	Yes	3
8—Male, 44	Right, bupivacaine, 5 mL	None	2 months	Yes	Increased	No	
9—Female, 75	Right, bupivacaine, 5 mL	None	1 month	Yes	Increased	Yes	1
10—Female, 29	Right, lidocaine, 3 mL	None	5 months	Yes	Unchanged	No	
11—Male, 45	Left, bupivacaine, 5 mL	None	2 months	Yes	Decreased	No	
12—Male, 80	Left, bupivacaine, 5 mL	None	1 month	No	Unchanged	No	
13—Female, 64	Right, bupivacaine, 5 mL	None	1 month	Yes	Decreased	No	
14—Female, 44	Right, bupivacaine, 5 mL	None	3 months	No	Unchanged	No	

Abbreviations: # = Patient sequential identification number; FU = Follow-up; N/A = Not applicable.

## Data Availability

The data presented in this study are available on request from the corresponding author due to patient privacy concerns.
